# Differentiation of Three *Centella* Species in Australia as Inferred from Morphological Characteristics, ISSR Molecular Fingerprinting and Phytochemical Composition

**DOI:** 10.3389/fpls.2017.01980

**Published:** 2017-11-21

**Authors:** Ali Alqahtani, Jun-Lae Cho, Ka Ho Wong, Kong M. Li, Valentina Razmovski-Naumovski, George Q. Li

**Affiliations:** ^1^Faculty of Pharmacy, University of Sydney, Sydney, NSW, Australia; ^2^Department of Pharmacognosy, King Saud University, Riyadh, Saudi Arabia; ^3^Discipline of Pharmacology, University of Sydney, Sydney, NSW, Australia; ^4^School of Science and Health, National Institute of Complementary Medicine, Western Sydney University, Sydney, NSW, Australia; ^5^South Western Sydney Clinical School, School of Medicine, University of New South Wales, Sydney, NSW, Australia

**Keywords:** *Centella* species, intersimple sequence repeats (ISSR), chemometrics, morphometrics, DNA fingerprinting, TLC

## Abstract

*Centella asiatica* is one of the popular herbs used for inflammatory and neural conditions. Its differentiation from similar species is currently lacking. The aims of this study were to differentiate the three closely related *Centella* species using methods based on morphological characters, genetic biodiversity, phytochemical compositions and antioxidant activities. According to the morphological characteristics, the collected samples were identified as three species: *C. asiatica, Centella cordifolia* and *Centella erecta* and clustered into three groups based on their morphometric variability. Dendogram constructed on the basis of the intersimple sequence repeats (ISSR) analyses were consistent with the morphological grouping. *Centella cordifolia* had the highest triterpene glycosides, phenolics and antioxidant capacity, followed by *C. asiatica*, then *C. erecta*, therefore, was genetically and chemically closer to *C. asiatica*, while *C. erecta* was distinctively different from them. The results confirm the occurrence of the closely related three species of *Centella* in Australia, and the differentiation among them can be achieved via the combination of morphometric, molecular and phytochemical methods. This first comparative botanical study on *Centella* species provides a foundation for further systematic study and medicinal development of *Centella*.

## Introduction

The genus of *Centella* L. comprises of more than 40 species (Mabberley, 2008; Roskov et al., 2013). The majority of these herbaceous perennial species are endemic to the Fynbos region of South Africa, and several species are also indigenous to tropical and subtropical regions of Asia, Africa, North and South America. *Centella asiatica* (L.) Urb., (Umbelliferae) is the most well-known species in this genus, and it was previously included in the *Hydrocotyle* genus. Since the 1980's botanical reports, phytochemistry and DNA molecular analyses reclassified *C. asiatica* in the *Centella* genus of the Umbelliferae family (Asakawa et al., 1982). Over the years, *Centella* species have been used in the treatment of skin disorders and as a memory enhancer. The main biological activities of *C. asiatica* have been attributed to the dominant pentacyclic triterpenoidal saponins, particularly asiaticoside, madecassoside, asiatic acid and madecassic acid. Phenolic compounds and flavonoids were also demonstrated to have specific bioactivities against neurotoxicity and oxidative stress-related disorders (Ponnusamy et al., 2008; Hussin et al., 2009). The anti-inflammatory and wound-healing properties of the *Centella* plant have been attributed to these chemical constituents (Orhan, 2012; Bylka et al., 2014).

*C. asiatica* and its very closely related species, including *Centella cordifolia* (Hook.f.) Nannf, and *Centella erecta* (L.f.) Fern., are found in Australia, mainly in tropical and subtropical places. The Australian native species, *C. cordifolia*, also named *Hydrocotyle cordifolia* (Hook.f.), was reported and described previously in botanical literatures, and is characterized with a distinctive leaf shape and non-spreading growing habit (Nannfeldt, 1924; Marchant et al., 1987; Harden, 1992). However, it is difficult to differentiate *Centella cordifolia* from *Centella asiatica* (Henwood and Hart, 2001; PlantNET, 2017). *C. erecta* is usually found in South America and in eastern and southern regions of North America. It has been listed by The Maryland Department of Natural Resources as a threatened and endangered species of the state of Maryland, and is believed to be introduced to Australia through plant nurseries (Liogier, 1995; Hogan and Belton, 2016). Although its morphology has been previously described by Liogier, *C. erecta* is easily confused with *C. asiatica* (Rumalla et al., 2010). These three species are difficult to distinguish morphologically, especially without a high level of taxonomy experience. The similarity of the three species has raised new concern on the potential adulteration of *C. asiatica* which is the only species approved for medicinal purpose.

*Centella* has been shown to occur in distinct morphotypes with differences in morphological characteristics, including leaf shape and inflorescence structure. The majority of *Centella* species and some of their allopatric varieties from South Africa show differences in leaf morphology, including leaf shape and the length of lamina and petiole, as well as the inflorescence and bract morphology (Schubert and Wyk, 1995, 1998, 1999; Schubert and van Wyk, 1997). *C. asiatica* displayed overall similarity to another species of *Centella*, such as those native to South Africa, including *Centella ternata, Centella umbellate, Centella calliodus* and *Centella lanata*, especially in the leaf morphology. However, certain morphological features, including leaf margin shape and the structure of the inflorescences, provide taxonomical standards to differentiate the species. *C. asiatica* showed indistinctly dentate leaf margin and stoloniferous habit compared to the serrate leaf margin and tufted, woody habit of other species (Schubert and van Wyk, 1995).

Morphological characterization, genetic study and chemical analysis play important roles in the accurate identification, proper safety and quality evaluation of medicinal plants, and impact on their pharmacological activities and respective clinical applications. The combination of these methods has been successfully applied in differentiating various medicinal plants (Borba et al., 2002; Casiva et al., 2002; Nielsen et al., 2003; Shi et al., 2009; Bhattacharyya et al., 2015). In contrast to *C. asiatica*, which has been thoroughly and extensively investigated, the morphological and genetic information, as well as chemical constituents of the other two species, are not comprehensively understood. The genetic variation of *C. asiatica* was reported in different samples from different localities in China (Zhang et al., 2012) and India (Prasad et al., 2014), but genetic characterization of closely related species is lacking. Although, previous studies suggested the influence of plant origin or growth conditions on the chemotype variation in the production of the active secondary metabolites (Schaneberg et al., 2003; Aziz et al., 2007; Randriamampionona et al., 2007; Zhang et al., 2009; Puttarak and Panichayupakaranant, 2012), there has not been an exhaustive study assessing this variation at the species level or its association to genetic variation. Since morphological characters are subject to environmental influences and could vary during different stages of plant development, the information from molecular marker fingerprinting combined with morphological data in assessing the genetic diversity and structure have been used for accurate species identification. In addition, extensive chemical evaluation in relation to genetic polymorphism should be carefully studied to ensure production sustainability.

Inter-simple-sequence repeat (ISSR) molecular marker, one of arbitrarily amplified dominant markers, has been successfully employed to assess genetic diversity in a variety of organisms including plants. ISSR polymorphisms represent the variation in the simple sequence repeat motifs (microsatellites) scattered in the genome. The amplification of this marker does not require previous information about genome sequence and, thus, can be produced using PCR primers composed of a few microsatellite units (Zietkiewicz et al., 1994). The reproducibility of multilocus patterns of ISSR-PCR makes it preferred over other molecular marker methods, such as Random Amplified Polymorphic DNA (RAPD).

Therefore, the aims of this study were to differentiate the three closely related *Centella* species using methods based on morphological characters, genetic biodiversity, phytochemical compositions and antioxidant activities. Specifically, this study employed: (1) morphology and ISSR molecular markers to investigate the level and pattern of taxonomy and genetic variability; (2) chemometrics coupled with morphometric analysis as a comprehensive method for differentiating the three species; and (3) correlation analysis of chemical components and antioxidant capacity among the three species. The information from this study will help to differentiate the *Centella* species with optimum chemical composition and antioxidant activity for proper plant selection and effective conservation.

## Materials and methods

### Plant collection and morphological examination

Twenty-nine plant samples representing the three species (*C. asiatica, C. cordifolia* and *C. erecta*) were collected, during a survey conducted in 2010, from different states in Australia, including New South Wales, Queensland, Victoria, Western Australia and Tasmania (Table 1), (Figure S2). Plant samples were collected during January–March, representing the summer season in Australia. The authenticity of the samples was done taxonomically, and all species were identified by Dr. George Li (The University of Sydney, Australia). The confirmation of plant species was calculated by matching samples with herbarium specimens reported by the National Herbarium of New South Wales (NSW), The Royal Botanic Gardens & Domain Trust, NSW, Australia, and The Royal Botanic Gardens, Kew, England. Voucher specimens were deposited at the Faculty of Pharmacy, University of Sydney, Australia. The collection permit was acquired under the National Parks and Wildlife Act 1974 from the Office of Environment and Heritage, NSW, Australia (License Number: S12282), the Department of Environment, Water and National Resources, SA, Australia (Permit Number: A26170-1), and the Department of Sustainability and Environment, VIC, Australia (Permit Number: 10006673).

**Table 1:** The sample code and collection location of *C. asiatica* (CA), *C. cordifolia* (CC) and *C. erecta* (CE) in Australia.

***C. asiatica***			***C. cordifolia***			***C. erecta***		
**Sample code**	**Collection location**	**Location *(Latitude, longitude)***	**Sample code**	**Collection location**	**Location *(Latitude, longitude)***	**Sample code**	**Collection location**	**Location *(Latitude, longitude)***
CA−1	New South Wales, Denistone	*−33° 48′ 3.05″, +151° 5′ 1.10″*	CC−11	Tasmania, Cradock	*−43°06′11.7″, +147°02′45.2″*	CE−6	New South Wales, Sydney	*−33°47′01.8″, +151°08′29.8″*
CA−2	New South Wales, Denistone	*−33° 47′ 54.43″, +151° 5′ 5.32″*	CC−15	Victoria, Mandurang	*−36°48′54.3″, +144°19′04.4″*	CE−8	New South Wales, Kingsgrove	*−33°55′58.2″, +151°05′37.9″*
CA−3	New South Wales, Sydney Olympic Park	*−33° 50′ 47.51″, +151° 4′ 51.46″*	CC−21	Victoria, Tyabb	*−38° 16′ 19.86″, +145° 13′ 15.27″*	CE−9	Western Australia, Busselton	*−33°42′06.7″, +115°19′24.2″*
CA−4	New South Wales, Sydney Olympic Park	*−33° 51′ 4.00″, +151° 4′ 30.56″*	CC−22	Victoria, Mount Evelyn	*−37°47′45.8″, +145°22′21.4″*	CE−92	Western Australia, Anniebrook	*−33°41′55.3″, +115°10′01.6″*
CA−5	New South Wales, Camperdown	*−33° 53′ 26.02″, +151° 11′ 8.76″*	CC−23	Victoria, Wantirna	*−37°51′14.4″, +145°12′40.1″*	CE−10	Western Australia, Busselton	*−33°41′52.1″, +115°21′35.4″*
CA−7	New South Wales, North Sydney	*−33°49′15.2″, +151°04′25.9″*	CC−24	Victoria, Anglesea	*−38°23′46.3″, +144°11′03.4″*	CE−102	Western Australia, Anniebrook	*−33°40′31.1″, +115°07′36.8″*
CA−13	Queensland, Mudgeeraba	*−28°05′59.4″, 153°19′27.1″*	CC−25	Victoria, Mount Evelyn	*−37°47′47.9″, +145°22′57.9″*	CE−12	Queensland, Mudgeeraba	*−28°05′58.7″, +153°19′21.3″*
CA−14	Queensland, Edge Hill	*−16° 54′ 11.62″, +145° 44′ 58.01″*	CC−26	Victoria, Wantirna	*−37°50′37.1″, +145°13′31.1″*	CE−20	New South Wales, Thornton	*−32°46′53.9″, +151°37′00.1″*
CA−16	New South Wales, Denistone	*−33°47′58.1″ +151°05′33.4″*				CE−27	New South Wales, Sydney	*−33°57′58.5″, +151°08′40.7″*
CA−17	New South Wales, Menai	*−34°00′57.1″ +151°00′56.4″*						
CA−18	New South Wales, Glenhaven	*−33°42′29.3″ +151°00′41.8″*						
CA−19	New South Wales, Dural	*−33°41′01.9″ +151°01′36.8″*						

Sixteen morphological traits were scored for each randomly collected plant sample. The morphological traits included quantitative characters: leaf surface area, leaf blade length [diameter from apex tip to leaf base (sinus point)], leaf blade width [diameter across the leaf base (sinus point)], number of leaf dentate or crenate, number of primary lateral veins, length of leaf petiole; and qualitative characters: color of leaf, texture of leaf, leaf blade shape, leaf margin shape, color of the bottom part of leaf petiole, amount of hair on leaf margins and petiole, color of flower, thickness of the stem, texture of the stem, and color of the stem. The principal components analysis (PCA) of morphological data was carried out using PLS_toolbox in the platform of MATLAB R2012b (The MathWorks, MA, USA; Applequist, 2006).

### Plant cultivation and extraction

All plant samples that were derived from vegetative multiplication of the mother plants, were cultivated in L60.5 × W25.5 × H22 cm pots, at the University of Sydney, NSW, Australia, with consistent irrigation and full sunlight. All samples (in the form of the aerial parts) were harvested in February and then air-dried in a ventilated room with controlled temperature (25 ± 2°C). The dried plant materials were stored at 25°C in an airtight container. The dried materials were ground into a fine powder and sieved through a No. 180 (180 μm size mesh) sieve. The plant powder was then extracted three times with methanol by sonication as follows: 7 g of the plant powder was mixed with 175 mL of methanol (1:25) and placed in an ultrasonic bath for 15 min and then filtered. The combined methanolic filtrate was concentrated down at 40°C with a vacuum rotary evaporator. The dried extract was weighed and stored at −20°C prior to the analysis.

### DNA extraction and PCR condition

Forty individuals of fresh young leaves of 20 plant pots, two samples from each plant pot, representing the three species, were extracted using a modified cetyltrimethylammonium bromide (CTAB) method (Allen et al., 2006). The quality and quantity of DNA were assessed by NanoDrop 1000 spectrophotometer (Thermo Scientific) and by electrophoresis on 1% (w/v) agarose gel. The purity of the extracted nucleic acid was estimated using the ratio of absorbance at 260 and 280 nm (A_260_/A_280_ ratio). The DNA samples were diluted in TE buffer to a concentration of 50 ng/μL and stored at −20°C prior to PCR amplification.

Two approaches were used to design the ISSR primers: DNA sequences from previous studies on *Centella* species, (available in NCBI Nucleotide database), were visually examined for the presence of microsatellite repeats and the primers were constructed accordingly. The second approach followed the primer design of published ISSR studies that were conducted in Centella species. Thirty-four ISSR primers, synthesized by Invitrogen™ Custom DNA Oligos (Thermo Fisher Scientific Inc.), were used to standardize the PCR conditions. Through several primer screenings, 14 ISSR primers, with good reproducibility and relatively producing informative polymorphic fragments, were selected for samples amplification (Table 2).

**Table 2:** Code, sequence and specific annealing temperature for each of the 14 selected primers.

**Primer code**	**Sequence (5′-3′)**	**PCR Annealing temp. (*Ta* °C)**
ISSR1	(AG)_8_C	57
ISSR4	(AC)_6_T	56
ISSR5	(CA)_8_AY	57
ISSR6	(AG)8TC	56.5
ISSR8	(CT)8GC	56.5
ISSR9	(CA)_8_AR	56.5
ISSR12	(GGAGA)_3_	56
ISSR17	(TG)_8_A	56
ISSR18	(TC)8C	57
ISSR24	(GA)8TC	57
ISSR25	(CA)8TA	56
ISSR26	(GA)8TT	56
ISSR27	(GT)8GA	57
ISSR29	(CA)8TC	57

*Y = C/G*.

*R = A/T*.

DNA amplification was performed in a 25 μL volume containing 20 ng genome DNA, 1 × Taq PCR buffer, 2.0 mM MgCl_2_, 0.2 mM of each dNTP, 0.75 μM primer, 0.6 units of Taq DNA polymerase and 0.2 μM of the single primer. The amplification reaction consisted of an initial denaturation step at 95°C for 3 min, followed by 43 cycles of 1 min denaturation at 94°C, annealing at *Ta*°C for 1:30 min, extension at 72°C for 2 min, and a final extension at 72°C for 5 min. The amplified products were resolved on 1% agarose gels, run at 100 V in 1.0 × TBE buffer and photographed using GelDoc™ EZ system (Bio-Rad Inc.). The amplifications were repeated twice and only clear repetitive bands were used in data analysis. Molecular weights were estimated using a 100 bp DNA Ladder.

### ISSR data analysis

Only strong and distinct electrophoretic bands were included in the statistical analysis and treated as diploid and dominant markers. The amplified bands were scored as a binary variable, present (1) or absent (0) of a particular band, for each primer. The parameters of population genetic variation were calculated using PopGene 32 (Yeh et al., 1997). Gene flow (*Nm*) and unbiased genetic distance among species population were calculated according to the Nei's similarity coefficient (Nei, 1972). Shannon's information index (*I*) (Shannon and Weaver, 1949) and Gene diversity (GST) was estimated according to the formula of Nei (1973). The distance matrix, based on Neighbor-Joining with pairwise distance matrix, was subjected to cluster analysis by the unweighted pair-group method with arithmetic average (UPGMA), and the dendrogram was constructed using the NTSYSpc-2.2 software package (Setauket, NY, USA). The relationships between the genetic diversity indices, chemotypic, and morphotypic parameters were estimated using the Pearson's correlation with SPSS 11.0 software. The goodness of fit, matrix correlation between the genetic distance and the morphological data matrices was calculated by Mantel's test (Mantel, 1967) (1,000 permutations) using the XLSTAT software (Addinsoft USA, New York, NY).

### TLC of phenolics and saponins components

Thin-layer chromatography (TLC) measurement was performed on pre-coated silica gel 60 plate from Merck (Catalog number: 105547; Darmstadt, Germany). The samples were dissolved in methanol at a concentration of 10 mg/mL and applied to a 10 × 10 cm TLC plate using CAMAG Linomat IV semi-automatic TLC sampler (Muttenz, Switzerland). Asiaticoside, madecassoside, chlorogenic acid, quercetin, rutin and kaempferol (50 μg/mL) were used as reference compounds. The application volume was 4 μL, with a bandwidth of 7 mm at an application speed of 77 nL/s, with a 2 mm space between bands, 10 mm from the side edges, 4 mm apart, and 15 mm from the bottom edge of the plate. The TLC plates were developed in a CAMAG chamber tank containing 8 mL of the mobile phase and pre-saturated for 30 min with the mobile phase at ambient temperature and humidity. Two solvent systems were used to detect either saponins or phenolics. The separation method of saponins was as previously described with some modifications (James and Dubery, 2011). The mobile phase mixture for saponins detection consisted of chloroform-glacial acetic acid-methanol-water (60:32:12:8 v/v). The mobile phase for the phenolics detection was optimized and consisted of toluene-ethyl acetate-formic acid water (3.5:12:1:0.5 v/v). The pre-loaded plates were developed to 8.5 cm for the saponins, and to 9.5 cm for the phenolics. After development, the plates were air dried for 5 min and sprayed with anisaldehyde-glacial acetic acid-methanol-sulfuric acid (0.5:10:85:5 v/v) for saponins detection, or Neu's reagent (solution A; consisting of 1% 2-aminoethyl diphenylborinate in methanol, solution B; consisting of 5% polyethylene glycol in ethanol) for phenolic detection. The plate was then incubated in an oven at 100°C for 5 min until the color of the bands on the plate appeared. The plates were observed under white light for saponins, or under UV light at 366 nm for the phenolics using a TLC visualizer (CAMAG REPROSTAR 3, Muttenz, Switzerland) and documented using a Canon EOS 700D digital camera with the aid of the EOS utility computer software (Canon USA, Inc.).

### Principal component analysis (PCA) of digitalised TLC profiles

For the PCA analysis of the TLC bands, the digitalization and transformation of the TLC plate images into data matrices was performed as described previously (Wong et al., 2014). To reduce the dimensions of the analyzed data, the TLC plate images were converted to 8-bit monochromatic grayscale using Matlab R2014b (The MathWorks, MA, USA). The number of pixels for each sample was set as 1,450. The pixel intensity was acquired in triplicate for each sample using the “Improfile” function of Matlab, and the mean was used to form the chromatographic data matrix (29 rows, representing the number of samples, and 1,450 columns, representing the number of pixels per sample). Several pre-processing parameters were applied to correct TLC band position which may occur due to any experimental variability of the TLC plates. The employed pre-processing functions were matrix smoothing (using Savitzky-Golay filter with a filter width of 17 and a zero order polynomial), peak alignment [using correlation optimized warping (COW) with slack of 10; segment length of 60 for saponins detection, and slack of 8; segment length of 55 for phenolics detection], standard normal variation (SNV) and mean centering. All previous parameters were optimized for optimum peaks fit and smooth baseline.

### Total phenolic content

Total phenolic content (TPC) was determined using a previously described protocol (Enayat and Banerjee, 2009). Briefly, 350 μg of the extracts were transferred to a 96-well plate and mixed with NaNO_2_ (5% w/v) for 5 min, and then AlCl_3_ (10% w/v) was added. After 6 min, 1 M NaOH was added to the mixture and incubated for 15 min. The absorbance was determined at 515 nm. Catechin was used as the standard for the calibration curve. All values were expressed as milligram catechin equivalents per gram of dried weight (mg CAE/g DW).

### Total saponin content

The total saponins content (TSC) was determined using a vanillin-glacial acetic acid colorimetric method as described in previous studies with some modifications (Chen et al., 2007; Yan et al., 2011). The plant extracts (625 μg) were transferred to test tubes and mixed with 0.2 mL freshly prepared 5% (w/v) vanillin-acetic acid solution. Perchloric acid (1.2 mL) was added and the mixture was incubated at 65°C for 15 min. After the mixture was cooled on ice, acetic acid was added to a final volume of 5 mL, then mixed and centrifuged. The absorbance of the supernatant was scanned at 557 nm by UV-VIS spectrophotometer. A saponin standard (containing 20–35% sapogenin, Sigma-Aldrich), in the range of 0.25–2 mg, was used as the standard equivalent, and TSC of the samples was calculated from the calibration curve and expressed as percentage saponins equivalents per gram of dried weight (% w/w).

### HPLC-PDA apparatus and chromatographic condition

Chromatographic analysis was performed on a Nexera X2 UHPLC system (Shimadzu, Kyoto, Japan), equipped with LC-30AD dual pumps, SIL-30AC autosampler with samples cooler and DGU-20A5R in-line vacuum degassing solvent delivery unit. For chromatographic separation, a reversed-phase C18 Aqua 5 μ 125A (250 × 4.60 mm) column (Phenomenex, NSW, Australia) coupled with a 1 mm Opti-Guard C18 pre-column (Choice Analytical Pty Ltd., NSW, Australia) was used. The aqueous mobile phase (solvent A) was 0.2% phosphoric acid in HPLC-grade water, while the organic phase (solvent B) was 0.2% phosphoric acid in 100% of HPLC grade acetonitrile. The gradient elution profile, based on the concentration of solvent B, was 0 min, 11%; 18 min, 13%; 33 min, 20%; 43 min, 25%; 50 min, 75%; 52 min, 88%; 64.5 min, 95%; 69 min, 11%; 82 min, 11%. The mobile phase flowed at a rate of 1 mL/min and the sample injection volume was 10 μL. The chromatograms were monitored at wavelengths of 204 nm for the four triterpenes, and 325 and 366 nm for chlorogenic acid and kaempferol, respectively, via SPD-M30A photodiode array detector. Shimadzu Labsolution software (Shimadzu, Japan) was used for chromatographic data processing.

The triterpenes, chlorogenic acid and kaempferol standard solutions were injected in the HPLC system and a calibration curve was created. A concentration of 4 mg/mL of plant samples was injected, and their components were identified according to the corresponding retention time and UV spectra of the authentic standards eluted under the same conditions. The quantitation of each compound and total contents was expressed as mg/g (dried weight) of the plant. Microsoft Excel 2013 (WA, USA) was used for statistical analysis and generating calibration curves.

### Antioxidant assays

#### 1, 1-Diphenyl-2-Picrylhydrazyl (DPPH) assay

The DPPH radical scavenging activity was determined as described previously (Sulaiman et al., 2011). Methanolic extracts (150 μg) were mixed with DPPH radical solution (0.24 mg/mL DPPH in methanol) and incubated for 30 min. The absorbance was determined at 515 nm. Trolox was used for the calibration curve. All values were expressed as micromolar trolox equivalents per gram of dried weight (μM TE/g DW).

#### 2, 2′-Azino-bis(3-Ethylbenzothiazoline-6-Sulphonic acid) (ABTS) assay

The ABTS radical scavenging activity was determined as previously described (Thaipong et al., 2006). The radical solution was produced by mixing equal volumes of 7.4 mM ABTS solution with 2.45 mM potassium persulfate and the mixture was stood in the dark for 12–16 h. The radical solution was diluted with 80% ethanol to an absorbance of 1.10 ± 0.02 at 734 nm to yield the working solution. The extracts (150 μg) were mixed with working solution (3 mL) and incubated in the dark for 30 min. The absorbance was determined at 734 nm. Trolox was used for the calibration curve. All values were expressed as micromolar Trolox equivalents per gram of dried weight (μM TE/g DW).

### Cupric ion reducing antioxidant capacity (CUPRAC)

The CUPRAC radical scavenging assay was determined as previously described (Apak et al., 2008; Chen et al., 2013). Briefly, 100 μL of plant extract was mixed with 1 mL CuSO_4_ (5 mM), 1 mL neocuproine (3.75 mM), 1 mL ammonium acetate (1 mM) and 1 mL distilled water and kept in the dark for 30 min in a 37°C water bath. The absorbance was measured at 450 nm. Results were expressed in micromolar of gallic acid per gram of dried weight (μM GAE/g DW).

### Statistical analysis of phytochemical constituents and antioxidant capacity

All values were expressed as mean ± SD. Data was analyzed by one-way analysis of variance (ANOVA). To satisfy ANOVA assumptions, data were transformed, followed by multiple comparisons tests (Tukey test) to estimate the significance of differences between groups. If the transformed data could not meet ANOVA assumptions, non-parametric analysis of variance (Kruskall-Wallis) test was performed.

## Results

### Morphological analysis

**Figure 1:**
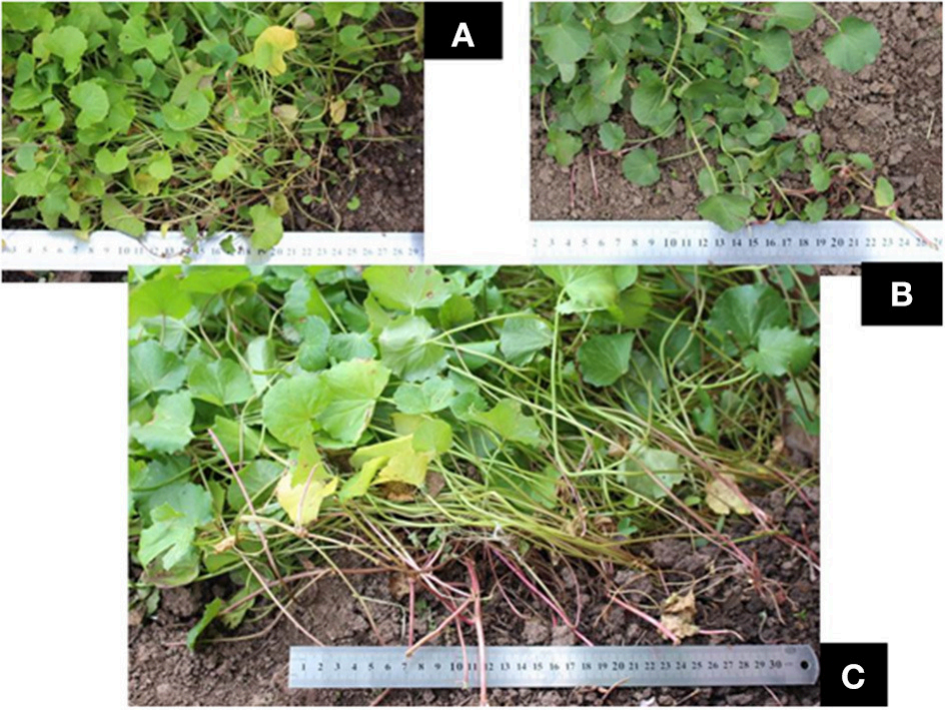
Cultivation of three *Centella* species. **(A)** *C. asiatica*; **(B)** *C. cordifolia*; and **(C)** *C. erecta*.

After cultivation at the same location, and comparative observation, each species showed distinct morphological characters (Table 3). The growing rate and habit were species-dependent (Figure 1). *C. erecta* tended to grow faster than the other species, with higher leaf mass observed per pot (data not shown). The stems of *C. cordifolia* and *C. erecta* were embedded very deeply in the soil, whereas the stem of *C. asiatica* grew along the surface. We suggest that the feature and growth pattern of the stem can be adopted as one of taxonomical characteristics to differentiate the three species. Furthermore, other morphological features of the leaf (leaf surface area, number of dentate or crenate and length of leaf petiole) were effective in differentiating these species. The morphometric analysis grouped all Australian samples into three main clusters, confirming the classification and the distribution of the three species in Australia.

**Table 3:** A morphometric dataset was obtained from 16 morphological variables based on the average of three readings from three randomly collected leaves.

**Sample ID**	**Species**	**Origin**	**CL**	**TL**	**LSA**	**LBL**	**LBW**	**LBS**	**LMS**	**NDC**	**NPV**	**LP**	**CBP**	**AH**	**CF**	**SS**	**TS**	**CS**
CA-1	*C. asiatica*	NSW	1	2	7.11	2.02	3.39	3	2	18.2	6.9	19.24	1	2	1	2	2	1
CA-2	*C. asiatica*	NSW	1	2	6.87	1.89	3.19	3	2	17.1	7	16.2	1	2	1	2	2	1
CA-3	*C. asiatica*	NSW	1	2	7.64	1.885	3.184	3	2	16.19	6.9	15.62	1	2	2	2	2	1
CA-4	*C. asiatica*	NSW	1	2	6.78	1.79	3.168	3	2	18.43	7.1	14.3	1	2	1	2	2	1
CA-5	*C. asiatica*	NSW	1	2	6.792	1.994	3.165	3	2	15.6	5.83	12.7	1	2	2	2	2	1
CE-6	*C. erecta*	NSW	1	1	31.6	4.79	6.5	3	2	16	7.17	24.25	1	3	2	1	1	1
CA-7	*C. asiatica*	NSW	1	2	6.88	1.88	3.2	3	2	17.9	6.9	14.9	1	2	1	2	2	1
CE-8	*C. erecta*	NSW	1	1	33.8	4.935	6.4	3	2	15	7.13	25.42	1	3	2	1	1	1
CE-9	*C. erecta*	WA	1	1	30.09	3.992	5.93	2	1	5.02	7.17	24.59	2	3	2	1	1	2
CE-92	*C. erecta*	WA	1	1	33.79	3.452	6.43	2	1	6.13	7.17	24.09	2	3	2	1	1	2
CE-10	*C. erecta*	WA	1	1	34.24	4.927	6.786	2	1	4	7.225	23.63	2	3	2	1	1	2
CE-102	*C. erecta*	WA	1	1	33.66	4.311	5.886	2	1	5.11	7.128	23.99	2	3	2	1	1	2
CC-11	*C. cordifolia*	TAS	2	3	15.37	3.14	4.451	1	2	19.72	7.08	22.56	1	1	1	1	1	2
CE-12	*C. erecta*	QLD	1	1	42.2	5.49	7.27	3	2	15.21	7.32	29.7	2	3	2	1	1	1
CA-13	*C. asiatica*	QLD	1	2	6.8	1.7	2.8	3	2	20	7	9	3	1	1	2	2	3
CA-14	*C. asiatica*	QLD	1	2	7.2	2.1	3.41	3	2	18.3	7	18	1	2	1	2	2	1
CC-15	*C. cordifolia*	VIC	2	3	15.11	3.23	3.98	1	2	20.4	7.2	20.5	1	1	1	1	1	2
CA-16	*C. asiatica*	NSW	1	2	6.9	2.1	3.51	3	2	17.3	7.1	15.9	1	2	1	2	2	1
CA-17	*C. asiatica*	NSW	1	2	7.83	2.04	3.37	3	2	18.44	6.93	19.38	1	2	1	2	2	1
CA-18	*C. asiatica*	NSW	1	2	6.82	2.2	3.08	3	2	15.89	6	15.1	1	2	2	2	2	1
CA-19	*C. asiatica*	NSW	1	2	7.8	2	3.3	3	2	14.1	7.1	15.9	1	1	1	2	2	3
CE-20	*C. erecta*	NSW	1	1	22.4	4.1	4.9	2	1	8	7.15	18	2	3	1	1	1	2
CC-21	*C. cordifolia*	VIC	2	3	15.32	3.5	3.78	1	2	20.9	7.3	19.5	1	1	1	1	1	2
CC-22	*C. cordifolia*	VIC	2	3	16.92	3.13	4.18	1	2	18.9	7.0	17.5	1	1	1	1	1	2
CC-23	*C. cordifolia*	VIC	2	3	15.44	3.47	4.11	1	2	19.66	7.1	18.6	1	1	1	1	1	2
CC-24	*C. cordifolia*	VIC	2	3	16.22	4.102	3.99	1	2	18.8	6.9	17.76	1	1	1	1	1	2
CC-25	*C. cordifolia*	VIC	2	3	12.1	2.98	3.21	1	2	17.8	6.98	18.9	1	1	1	1	1	1
CC-26	*C. cordifolia*	VIC	2	3	11.9	2.73	3.82	1	2	18.4	7	19.5	1	1	1	1	1	1
CE-27	*C. erecta*	NSW	1	1	32.7	4.19	6.2	3	2	18.1	7.17	21.55	1	3	2	1	1	1

*CL; Color of leaf: 1, Green; 2, Dark green*.

*TL; Texture of leaf: 1, surface and hard texture: 2, Rough surface and soft texture; 3, Rough surface and hard texture*.

*LSA; Leaf surface area; cm^2^*.

*LBL; Leaf blade length–diameter from apex tip to leaf base (Sinus point); cm*.

*LBW; Leaf blade width–diameter across the leaf base (Sinus point); cm*.

*LBS; Leaf blade shape: 1, Cordate; 2, Orbicular; 3, Reniform*.

*LMS; Leaf margin shape: 1, Entire with little dentate; 2, Dentate*.

*NDC; Number of dentate or crenate*.

*NPV; Number of primary lateral veins*.

*LP; Length of leaf petiole*.

*CBP; Color of the bottom part of leaf petiole: 1, Pink; 2, White; 3, Green*.

*AH; Amount of hair on leaf margins and petiole: 1, High; 2, Medium; 3, Low*.

*CF; Color of flower: 1, Red to purple; 2, White to Light Pink*.

*SS; Thickness of the stem: 1, Thick; 2, Thin*.

*TS; Texture of the stem: 1, Hard; 2, Soft*.

*CS; Color of the stem: 1, Reddish to Pink; 2, Pink to dark brown; 3, Brown to green*.

Upon close investigation, we found that the morphological characteristics of the three *Centella* species were distinct. The stem of both *C. cordifolia* and *C. erecta* displayed a thick and hard texture compared to *C. asiatica* which was thin and fragile. The leaves of *C. asiatica* and *C. cordifolia* were smaller in size (6.78–7.83 and 11.90–16.92, cm^2^, respectively) than *C. erecta* (22.40–42.20, cm^2^) and had a cordate blade shape which was more dentate or crenate in leaf margin, while *C. erecta* had reniform-shaped leaves with smooth-glossy texture and little dentate in the margin.

**Figure 2:**
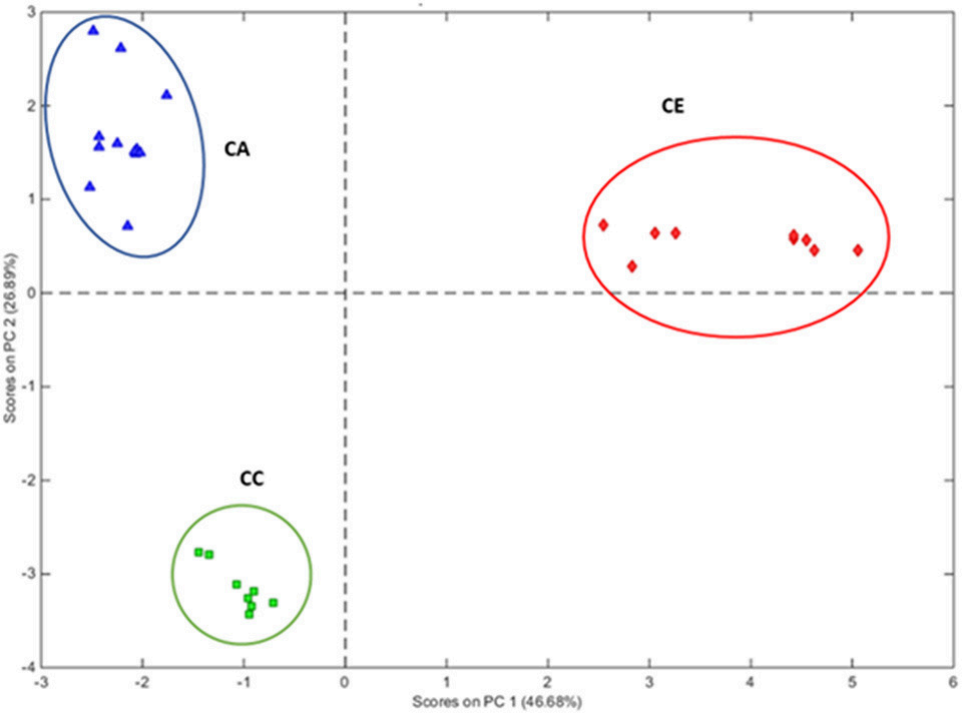
PCA multivariate analysis of morphometrics grouping the 29 *Centella* plant samples according to their morphological characters. Clusters with blue, green, and red circles represent CA, *C. asiatica;* CC, *C. cordifolia*; and CE, *C. erecta*; respectively.

PCA was performed based on the variability of morphological characters of all individual plants of the three *Centella* species (Table 3). The results have shown that multivariate method differentiated the three *Centella* species, where clustering analysis based on the 16 morphometric parameters revealed that the morphological traits differed among the three species. All samples were grouped into three main clusters as per the species nomenclature that was given to each sample upon the identification (Figure 2).

For the corresponding eigenvector value, the first component accounted for 46.68% of the total morphometric variation, and was equated to leaf surface area (12.58%), leaf blade width (12.43%), and leaf blade length (10.28%), which were collectively greater than the eigenvectors of the total variation of morphological parameters in the second PCA component. The second component accounted for 26.89% of the total variation and grouped the samples according to the leaf color (21.14%) and leaf blade shape (20.31%).

Upon microscopic observation (Figure S1), six to eight vascular bundles were observed in the stems of *C. asiatica* and *C. cordifolia*. However, the vascular bundles on the stem of *C. erecta* were found to be more than other species; 8–10 vascular bundles were dependent on the size of the stem. The old stem from secondary growth showed that the periderm layers of the stem consisted of 2–3 layers, including phellem (cork), phellogen (cork cambium) and phelloderm, for both *C. asiatica* and *C. erecta*, with red anthocyanidin pigmentation next to the phelloderm. However, the periderm was mainly one layer for *C. cordifolia*, with absence of anthocyanidin.

### ISSR molecular analysis

From the 34 arbitrary ISSR primers, 14 informative primers, which generated consistent, unambiguous and reproducible fragments, were selected for the estimation of genetic similarities among samples (Table 2). One hundred and fifteen bands, ranging in size from 200 to 2,200 bp, were generated, and 101 bands were polymorphic (87.8%). The highest polymorphic bands were generated from primers (TC)_8_C, (TG)_8_G, (GA)_8_TT, (GT)_8_GA and (CA)_8_TC which produced 7–9 polymorphic bands. (GA)_8_TC produced three bands and was the lowest among the tested primers. All tested primers were 3′-anchored primers and were successful in generating reproducible amplification bands. Anchoring the primer at its 3′ end reduces the number of sequences that have homology to the primer and results in clear and distinct fragment bands (Parsons et al., 1997).

**Figure 3:**
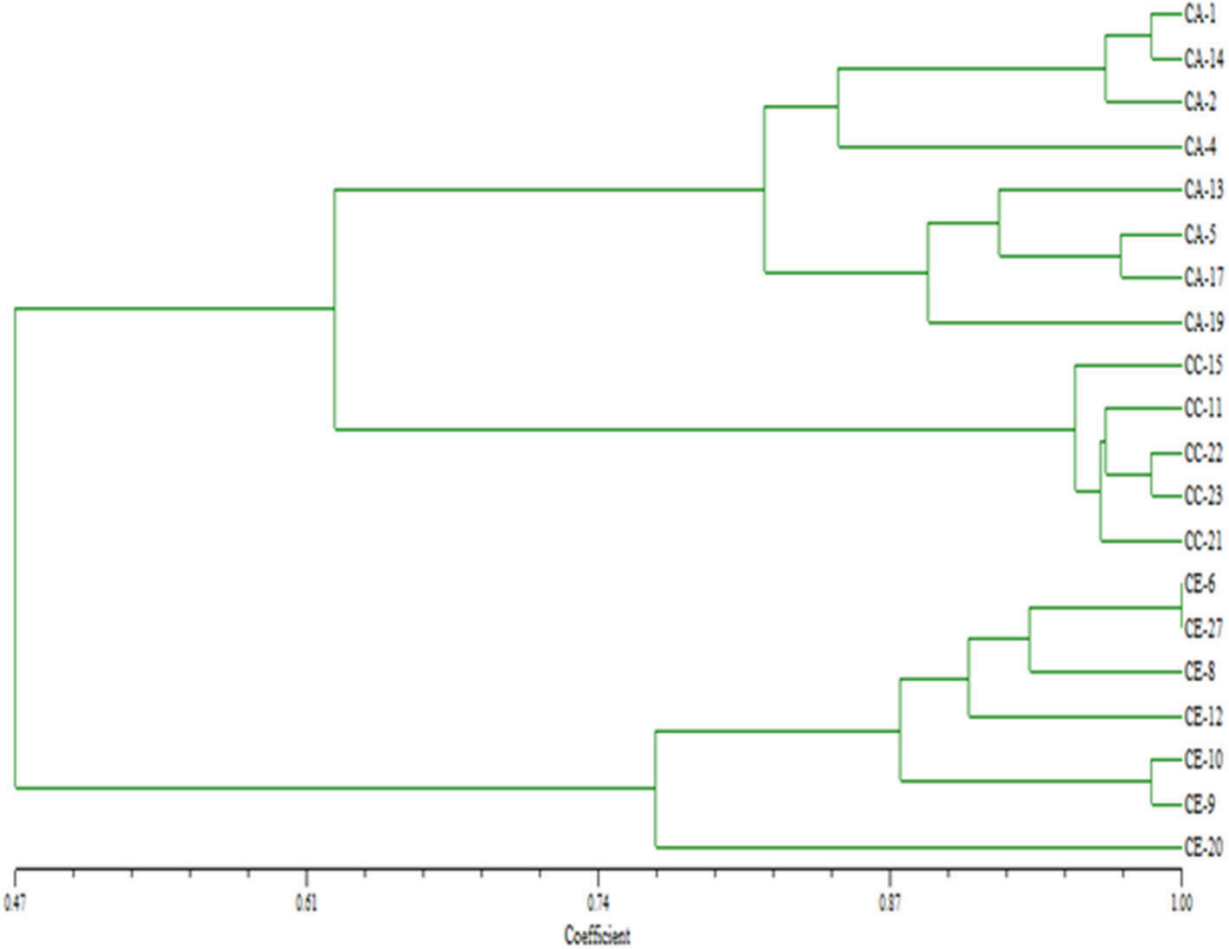
UPGMA dendogram based on Nei's genetic distances among the populations of three *Centella* species. CA, *C. asiatica*; CC, *C. cordifolia*; and CE, *C. erecta*.

**Figure 4:**
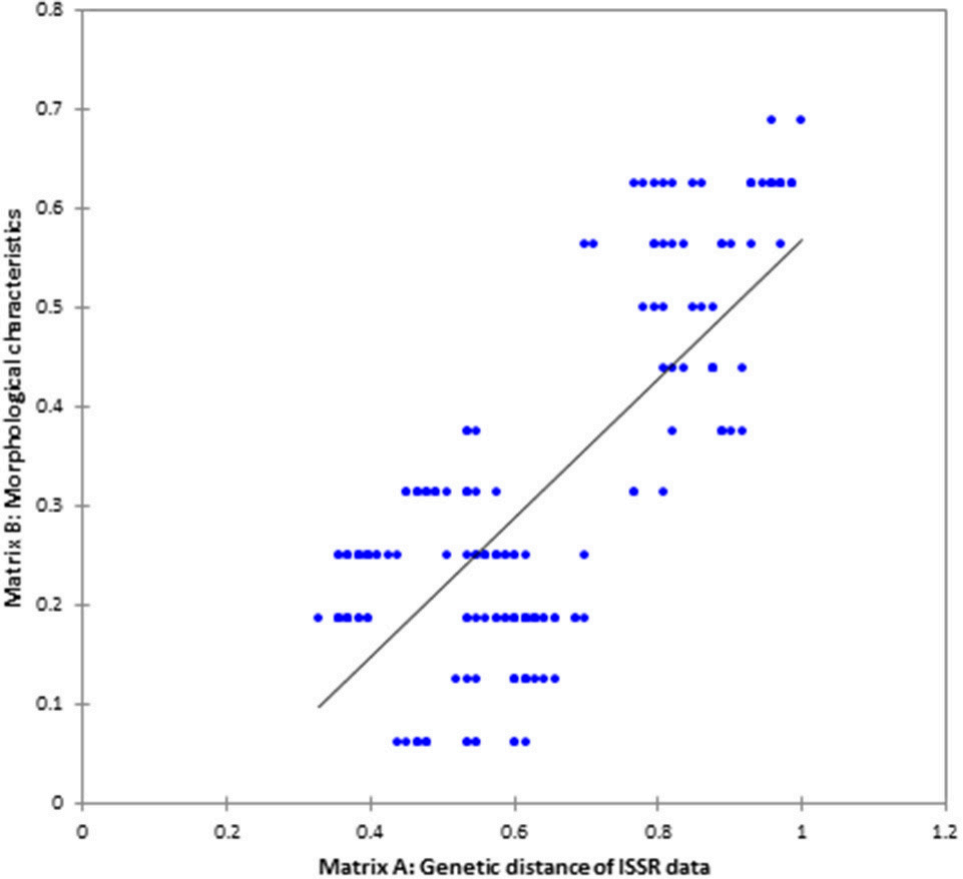
The correlation between ISSR genetic diversity and morphological trait of *Centella* samples as revealed from Mantel's-test (*p* < 0.01), two-tailed test.

Genetic diversity parameters were calculated based on the ISSR polymorphism. The UPGMA dendrogram based on Nei's genetic distance clustered all 20 populations into three groups at a similarity coefficient of 0.47–1.00, indicating richness of genetic diversity. *C. asiatica* and *C. cordifolia* were firstly clustered at similarity of 0.61, and then with *C. erecta* at 0.47 (Figure 3). It showed *C. asiatica* and *C. cordifolia* were genetically closer, but distinct from *C. erecta*. Among population of *C. erecta*, CE-6 and CE-27 were almost identical which was consistent with morphology. ISSR molecular traits was significantly correlated to the grouping pattern based on morphological characters as revealed from the Mantel's correlation (*r* = 0.75841), *P* < 0.01 (two tailed test), with performing 1,000 random permutations (Figure 4). The texture of the leaf (TL) showed high positive correlation to the genetic diversity indices as revealed from Pearson's correlation (H; *r* = 0.960, I; *r* = 0.946). Similarly, leaf surface area (H; *r* = 0.815, I; *r* = 0.838), leaf blade width (H; *r* = 0.837, I; *r* = 0.857), and the amount of hair on the leaf margins and petiole (H; *r* = 0.940, I; *r* = 0.932) were highly, negatively correlated to the genetic diversity indices (Table S1).

### PCA analysis of TLC chemical profiles

**Figure 5:**
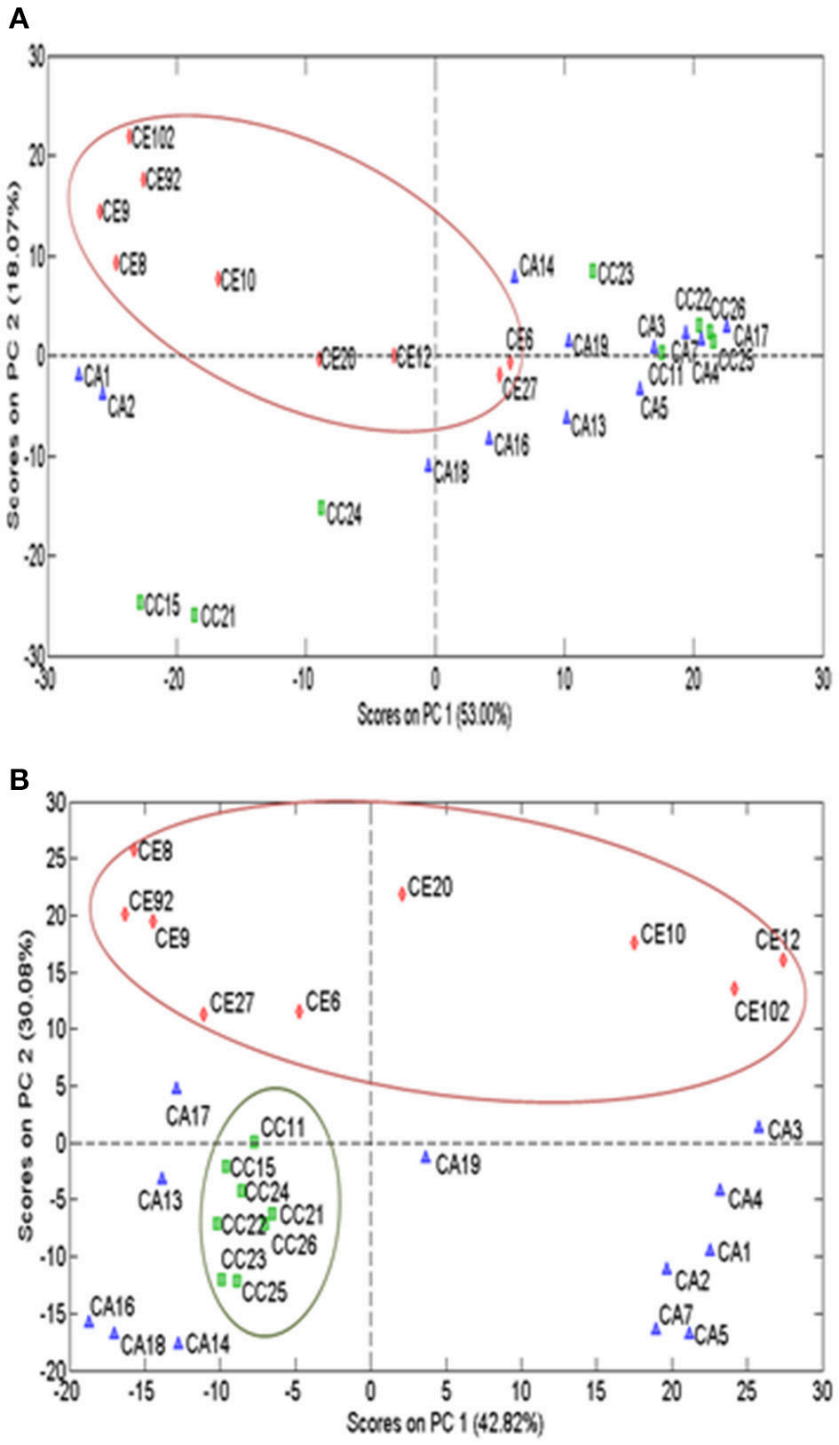
PCA scores plot of processed TLC data matrix of saponins and phenolic profiles of *Centella* species. Saponins **(A)** and phenolics **(B)** PCA clustering of three *Centella* species (*C. asiatica*: blue, *C. cordifolia*: green and *C. erecta*: red.

All chromatographic profiles obtained from the two TLC systems were subjected to the subsequent classification. After the application of pre-processing methods (smoothing, baseline removal and peak alignment), the data matrix was subjected to column centering, normalization and standard normal variate (SNV). Based on the TLC saponins profile, *C. erecta* samples was separated from other species (Figure 5A). Further TLC analysis of the flavonoids, separated *C. erecta* and *C. cordifolia* into two clusters, suggesting that these species had distinct chromatographic characteristics (Figure 5B).

### Total phenolic and saponin content

**Figure 6:**
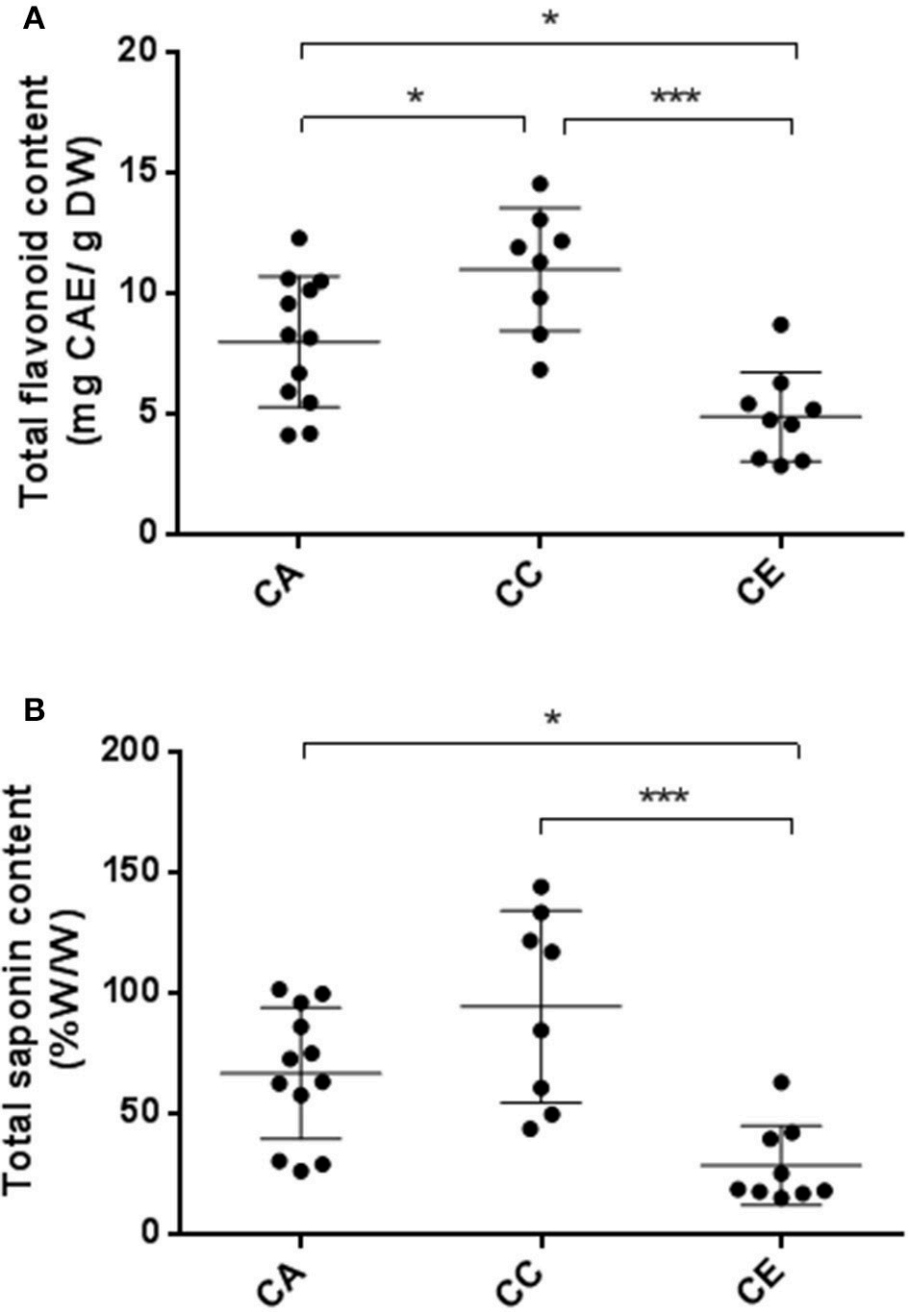
Variability of TPC **(A)** and TSC **(B)** between the three *Centella* species. CA, *C. asiatica;* CC, *C. cordifolia;* and CE, *C. erecta*. TPC data presented as milligram catechin equivalents per gram of dried weight (mg CAE/g DW). TSC data expressed as percentage saponins equivalents per gram of dried weight (% w/w). The error bar was calculated from SD. Tukey method, ANOVA ^*^p < 0.05; ^***^p < 0.001.

Figure 6 illustrates the TPC and TSC of the 29 selected samples from the three species. TPC demonstrated some variation in the range between 2.84 ± 0.15 and 14.53 ± 0.78 mg CAE/g DW, averaging 7.98 ± 0.29, 10.99 ± 0.30, and 4.87 ± 0.23 for *C. asiatica, C. cordifolia* and *C. erecta*, respectively. The results showed that *C. cordifolia* methanolic extract has significantly greater TPC than *C. erecta* and *C. asiatica* (*p* < 0.001 and *p* < 0.05, respectively). Furthermore, *C. erecta* exhibited lower TPC than *C. asiatica* (*p* < 0.05). Further analyses of the TSC of *Centella* samples showed that values varied from 14.85 ± 2.60 to 143.98 ± 3.54%, averaging 66.55 ± 3.67, 94.23 ± 3.02, and 28.37 ± 3.10% for *C. asiatica, C. cordifolia* and *C. erecta*, respectively. The overall results showed that the *C. erecta* methanolic extract had significantly lower TSC than *C. asiatica* and *C. cordifolia* (*p* < 0.05 and 0.001, respectively). However, no significant difference in TSC was found between *C. asiatica* and *C. cordifolia*.

### HPLC-PDA quantification of triterpenes, chlorogenic acid and kaempferol

**Figure 7:**
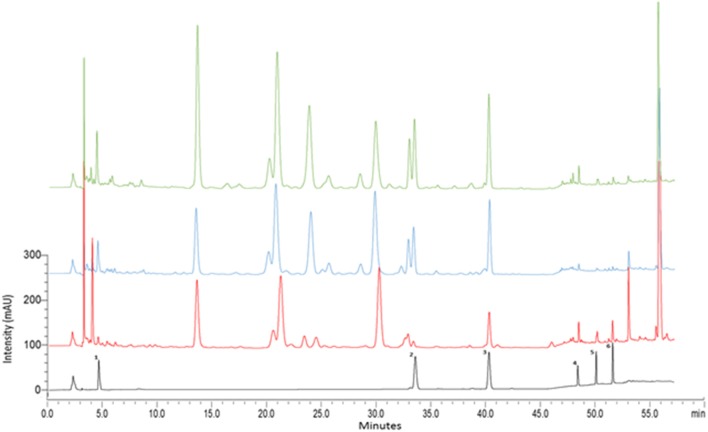
HPLC–PDA chromatograms of methanolic extracts of *Centella* species. Three selected samples representing *C. cordifolia* (CC21: green), *C. asiatica* (CA7: blue), *C. erecta* (CE10: red) at 240 nm. 1, chlorogenic acid; 2, madecassoside, 3, asiaticoside, 4, kaempferol, 5, madecassic acid; 6, asiatic acid.

The developed HPLC-PDA method was validated in terms of linearity, accuracy, precision, limits of detection (LOD) and limits of quantitation (LOQ) according to the International Conference on Harmonisation (ICH) guidelines (ICH Harmonised Tripartite Guideline, 2005) (data not shown). HPLC-PDA investigated the presence of the triterpene glycosides (madecassoside and asiaticoside) and aglycones (madecassic acid and asiatic acid), chlorogenic acid and kaempferol in the three species. By comparing the retention time and the UV absorbance spectra of the reference standards, all compounds were identified in the three *Centella* species as shown in Figure 7. The identities of the chemical compounds were confirmed by HPLC-HESI-MS analysis (data not shown).

**Figure 8:**
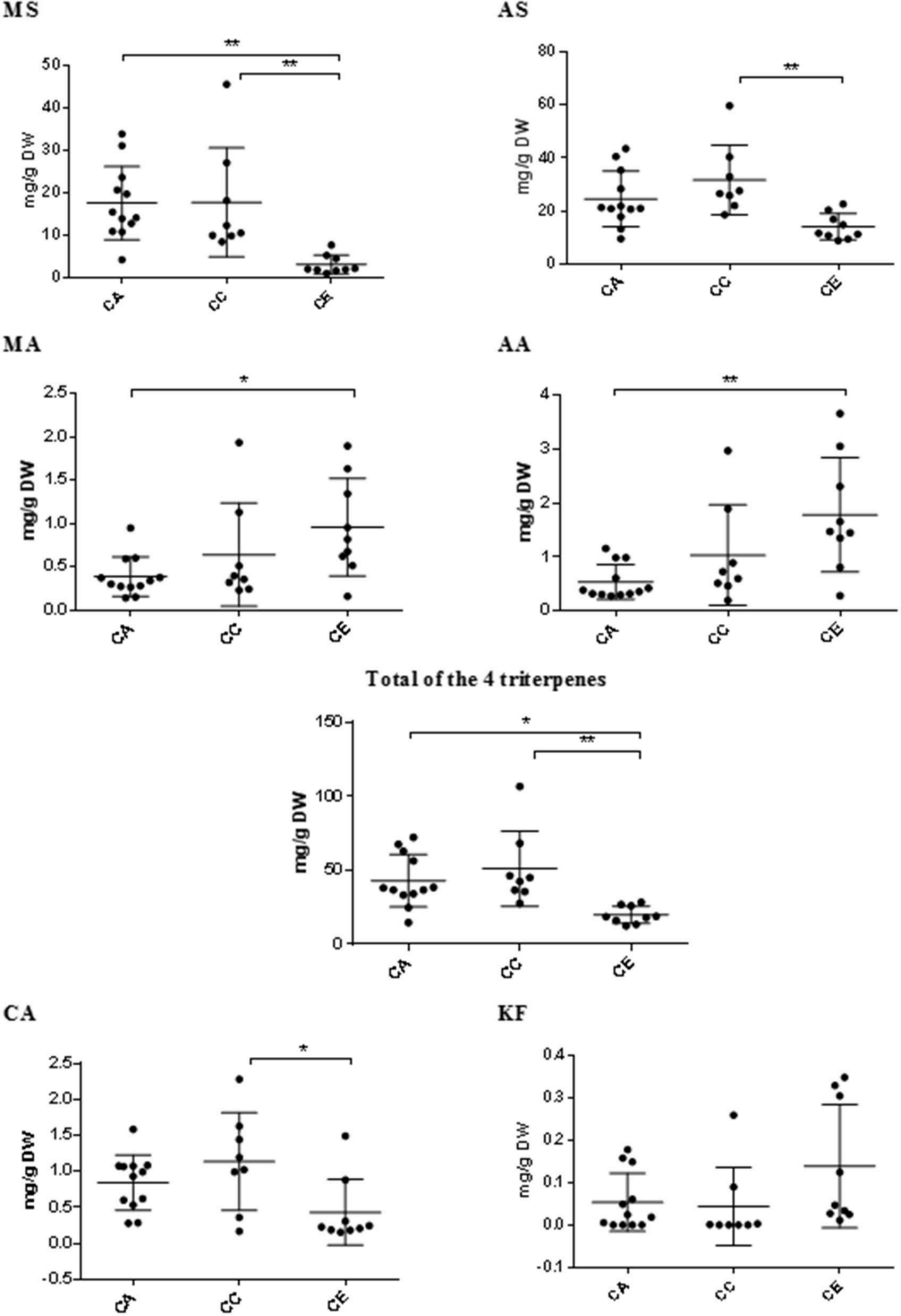
Variations in triterpene compounds and phenolics between *Centella* species. Triterpene glycosides (MS, madecassoside and AS, asiaticoside) and aglycones (MA, madecassic acid and AA, asiatic acid); CA, chlorogenic acid; and KF, kaempferol; content vary among the three *Centella* species by HPLC-PDA; CA, *C. asiatica;* CC, *C. cordifolia*; and CE, *C. erecta*. Data presented as mg/g of the dry weight (DW) with error bar calculated SD. Tukey method, ANOVA ^*^p < 0.05; ^**^p < 0.01.

The results from HPLC quantification of the target triterpene and phenolic compounds are summarized in Figure 8. The average content of madecassoside, asiaticoside, total triterpenes and chlorogenic acid in *C. erecta* (3.00 ± 2.22, 14.04 ± 4.94, 19.79 ± 5.89, and 0.43 ± 0.46 mg/g DW, respectively) were significantly less than what was observed from *C. cordifolia* (17.68 ± 12.88, 31.73 ± 13.14, 51.08 ± 9.00, and 1.14 ± 0.68 mg/g DW, respectively). Madecassoside and total triterpenes contents in *C. erecta* (3.00 ± 2.22 and 19.79 ± 5.89 mg/g DW, respectively) were found to be significantly lower than *C. asiatica* (17.54 ± 8.67 and 42.97 ± 17.81 mg/g DW, respectively). The content of triterpene aglycones in *C. asiatica*, including madecassic acid and asiatic acid (0.39 ± 0.23 and 0.54 ± 0.32 mg/g DW, respectively), were significantly lower than what was measured in *C. erecta* [0.96 ± 0.56 and 1.78 ± 1.06 mg/g (DW), respectively].

Although there was high variability of kaempferol content within and among the analyzed species, no significant differences were observed between the *Centella* species (*p* < 0.05).

### Antioxidant activities

**Figure 9:**
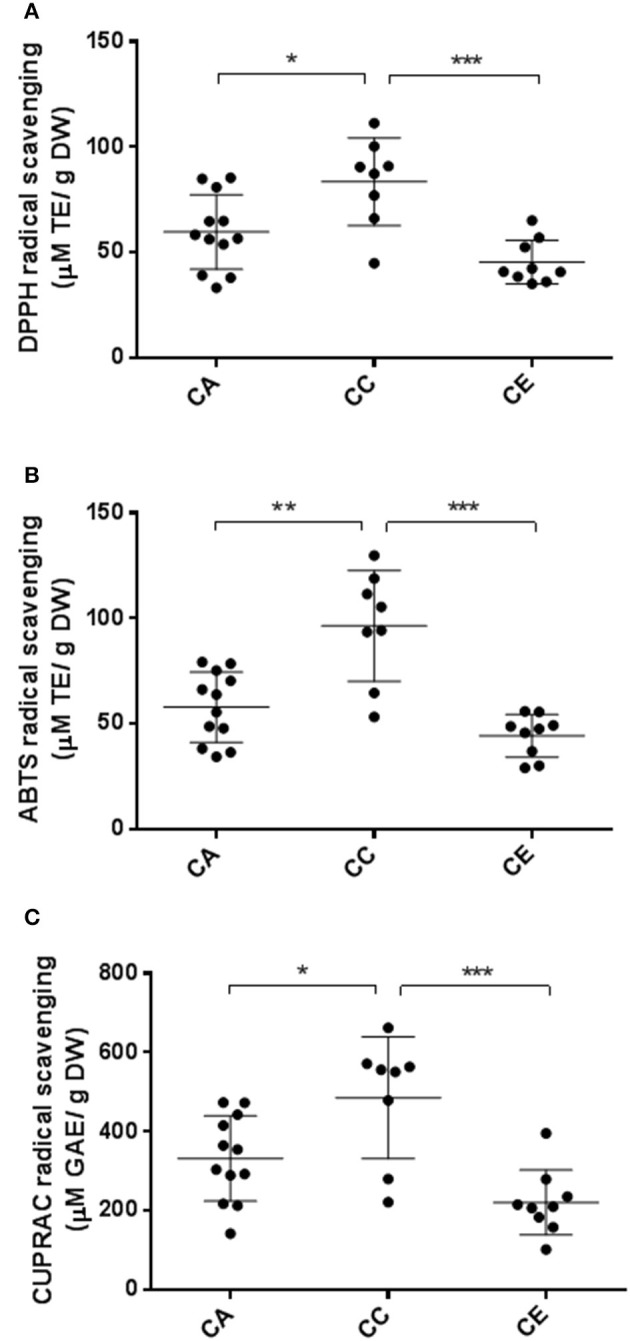
Variations in antioxidant capacity of the three *Centella* species. CA, *C. asiatica*; CC, *C. cardiofolia*; and CE, *C. erecta*. DPPH **(A)** and ABTS **(B)** data were expressed as micromole Trolox equivalents per gram of dried weight (μM TE/g DW). CUPRAC **(C)** data are presented as micromole gallic acid equivalents per gram of dried weight ((μM GAE/g DW). The error bar was calculated from SD. Tukey method, ANOVA ^*^p < 0.05; ^**^ *p* < 0.01; ^***^p < 0.001.

The total antioxidant capacities of all *Centella* samples as determined by ABTS, DPPH and CUPRAC assays are shown in Figure 9. The results indicated that the DPPH radical scavenging activity varied from 33.31 ± 3.88 to 111.36 ± 2.93 μM TE/g DW, averaging 59.76 ± 3.80, 83.60 ± 3.94, and 45.44 ± 3.16 μM TE/g DW for *C. asiatica, C. cordifolia* and *C. erecta*, respectively. For ABTS radical scavenging activities, the values varied from 29.15 ± 2.8 to 119.0 ± 1.3 μM TE/g DW, averaging 57.94 ± 2.55, 96.46 ± 2.4 and 44.38 ± 2.28 μM TE/g DW for *C. asiatica, C. cordifolia* and *C. erecta*, respectively. The antioxidant capacity as revealed by CUPRAC radical scavenging assay ranged from 102.27 ± 10.00 to 662.27 ± 7.56 μM GAE/g DW, averaging 332.04 ± 9.72, 485.65 ± 10.37, and 220.97 ± 8.99 μM GAE/g DW for *C. asiatica, C. cordifolia* and *C. erecta*, respectively.

Consistent with literature, total flavonoids values were highly correlated (*p* < 0.01) with DPPH, ABTS and CUPRAC radical scavenging activities, with *r*-values of 0.884, 0.823 and 0.883, respectively.

## Discussion

In this study, we investigated morphological characteristics of three different *Centella* species found in Australia for the first time. The samples of *C. cordifolia, C. asiatica* and *C. erecta* shared similar morphological features of *Centella* genera, such as fleshy leaves with venation palmate, widened and sheathing petiole bases and trailing stems. Based on previous phylogenetic study of Australian Apiaceae, it was concurred, to a certain extent, that morphological diversity of the leaf characteristics can determine the resolution of the genetic affinities among and within the genus (Henwood and Hart, 2001). Although the differences between the species could not be ascertained for the genus *Centella* in previous taxonomic revisions (Schubert and van Wyk, 1995), this study showed that plant leaf morphology, together with the features and growth pattern of the stem, are the main taxonomical determinants for species differentiation. Our results provide evidence that the three species can be identified clearly although they are very similar in morphology. No intermediates or hybrid between these three species were found as indicated previously by The NSW Plant Information Network System of Royal Botanic Gardens and Domain Trust (PlantNET, 2017).

Genetic diversity among those species was also explored in this study using ISSR molecular markers. The results demonstrated that *C. cordifolia* samples were genetically closer to *C. asiatica* population; and *C. erecta* was the most genetically distinct. The genetic diversity was highly correlated to the variability in the morphological trait among the samples, especially for the leaf texture and the amount of hair on the leaf margins and petiole, indicating that genetic factors have considerable impact on the evolution of the morphological characteristics of this plants. The dendrogram (Figure 3) is a reliable fingerprint of genetic diversity and relationship of the three species and population, which has advantages of simple, accurate and straightforward identification.

Numerous reports attributed the high degree of variability in phytochemical composition among *C. asiatica* samples to environmental or geographical factors (Randriamampionona et al., 2007; Zhang et al., 2009; Devkota et al., 2010; Thomas et al., 2010; Prasad et al., 2014). To the best of our knowledge, there are no reports evaluating such variability at the species level. We hypothesized that genetic variability among Australian *Centella* species determined the phytochemical profile and antioxidant potency. To support our hypothesis, we assessed the grouping patterns of the three Centella species based on ISSR molecular markers and morphological traits with the aid of multivariate analysis, in which all the three species were classified accordingly (Figures 2, 3), and compared these patterns to the phytochemical and antioxidant profiles. We found that a considerable variation exists in the composition of saponins and phenolics, as well as antioxidant capacity, which is consistent with the morphological and genetic clustering patterns of the three species. Phytochemical profiles were significantly different between *C. erecta* and *C. cordifolia*. As illustrated in Figure 6, *C. cordifolia* was shown to have the highest content of total saponins and phenolics compared to *C. erecta* and *C. asiatica*. On the contrary, *C. erecta* had considerably lower phenolic and saponin content among the three species, and statistically different in comparison with *C. cordifolia*.

The triterpene glycosides (madecassoside and asiaticoside) and their relevant aglycones (madecassic acid and asiatic acid) have been identified in *C. asiatica* and *C. erecta* using LC-UV, LC-MS and NMR (Rafamantanana et al., 2009; Rumalla et al., 2010). Here, the presence of these compounds in the methanolic extract of *C. cordifolia* using HPLC-PDA methods has been shown for the first time. Chlorogenic acid was reported previously by LC-UV and LC-MS in different *Centella* species, including *C. asiatica* and *C. glabrata* (Gray et al., 2014; Maulidiani et al., 2014). Moreover, kaempferol and its derivatives were reported in *C. asiatica* and in other plant species of the same family (*Hydrocotyle bonariensis* and *Hydrocotyle sibthorpioides*) (Maulidiani et al., 2012, 2014). However, there are no reports of these compounds in both *C. erecta* and *C. cordifolia*. Even though chlorogenic acid and kaempferol derivatives, including isochlorogenic acids, kaempferol-3-*O*-β-D-glucuronide and kaempferol-3-*O*-β-D-glucoside, were isolated from *C. erecta* (Rumalla et al., 2010; Gray et al., 2014), this study identifies the presence of chlorogenic acid and kaempferol compounds in *C. erecta* and *C. cordifolia* for the first time.

Interestingly, the variation pattern of the results obtained from the TSC colorimetric determination of the three species (Figure 6B) was consistent with the quantification results of the total content of the four triterpene glycosides and aglycones (asiaticoside, madecassoside, asiatic acid and madecassic acid) obtained by HPLC-PDA analysis (Figure 8). In both methods, *C. erecta* samples had the lowest content of total saponins and sapogenins compared to the other two species. This indicates that the colorimetric method for total saponins estimation could be applied to estimate the total content of those compounds, and hence, can be considered as a rapid and inexpensive method to predict the *Centella* species (such as *C. erecta*).

The antioxidant capacity showed similar tendency to the results of total phytochemical analysis. We found that the antioxidant effects of *C. cordifolia*, measured by DPPH and ABTS assays, was significantly higher than *C. asiatica* and *C. erecta*. From the CUPRAC assay, the radical scavenging activities of *C. cordifolia* were not significantly different to the *C. asiatica* samples, but the difference between *C. cordifolia* and *C. erecta* in this assay was significant (*P* < 0.01). For the correlation between ISSR molecular parameters and the plant's phytochemical contents, the highest correlation, based on Pearson coefficient, was observed between the effective number of alleles and TPC and ABTS antioxidant capacity (*r* = 0.703 and 0.680, respectively) (*P* < 0.01) (Table S1).

Although the variability in the antioxidant potency was found at the species level, a wide difference can be seen within samples from the same species. We found that the differences between the highest and lowest values in the antioxidant capacity within the *C. asiatica* samples was 61.01, 56.61, and 69.93%, *C. cordifolia* samples was 59.62, 58.93, and 66.55% and *C. erecta* samples was 46.00, 47.86, and 74.17%, for DPPH, ABTS and CUPRAC assays, respectively. This was in agreement to previous studies, whereby DPPH antioxidant capacity values ranged from 32.6 to ~50 μM TE /g DW in *C. asiatica* (Wong et al., 2006; Ariffin et al., 2011). The variability in the antioxidant effects of *C. asiatica* in the literature could be due to the influence of seasonal changes or geographical factors. In a previous study, we found that seasonal changes influence chlorogenic acid and kaempferol contents of *C. asiatica*, and those compounds are well-known for exerting antioxidant properties (Alqahtani et al., 2015).

A previous study demonstrated that the diversity observed at the genetic level within six populations of *C. asiatica* with different leaf morphology was shown to be linked to the diversity of the asiaticoside content (Rakotondralambo et al., 2013). This could be attributed to genetic factors, regardless of the origin or environmental influences, as concluded in the previous studies (Randriamampionona et al., 2007; Rakotondralambo et al., 2013). The PCA analysis of TLC separated *C. erecta* and *C. cordifolia* samples via their chemical fingerprint. The data matrices obtained from the TLC profile of the flavonoids classified only *C. erecta* samples, while the matrices obtained from the TLC profile of saponins generated a better classification of both *C. erecta* and *C. cordifolia* species. It has been reported that *C. asiatica* from Madagascar origin contained 3–7 times higher asiaticoside concentration and achieved higher radical scavenging activity comparing with those from India (Rouillard-Guellec et al., 1997). The findings observed from our study was in agreement with the previous studies in which the relatively high correlation of morphological traits, chemical contents and antioxidant capacity to the genetic diversity suggest the wide distribution of a high number of genes over the plant genome that control the phenotypic composition including secondary metabolites (Thomas et al., 2010).

The correlation between the chemical components and the genetic information suggest that the basis of variation in the phytochemical composition depends mainly on the genetic background that can be predicted from the morphological characteristics of the species. Differences in secondary metabolite contents in *C. asiatica* from different geographical regions was reported previously (Devkota et al., 2010). The genetic background might be significantly important for inducing fundamental variation in the secondary metabolites. The variation in triterpenoid contents in *C. asiatica* samples growing in different regions of Madagascar was shown previously, and genetic factors was suggested to attribute to the chemical differences (Randriamampionona et al., 2007). Moreover, the diversity in triterpene profile, especially asiaticoside and madecassoside levels, in Indian varieties was suggested to be genetically determined rather than due to external influences (Thomas et al., 2010; Prasad et al., 2014).

There is some limitation to the methods of the current study. In Australia, there are strict regulations for collecting plant materials. *C. cordifolia* is an endemic species in Australia which has very scattered distribution, and *C. erecta* is only available in nurseries, which makes it practically difficult to collect more samples. The ISSR method does not provide information on phylogenetic relationship of species. Therefore, future work on *Centella* species, including Australian species and other species of South Africa, should employ a larger sample number, latest methodology such as LC-MS, DNA sequencing, high performance TLC to understand their phylogenetic relationship, and potential application in medicinal development.

The morphometric method is efficient and convenient in the differentiation of the three species, particularly using the shape and size of leave, the size and habitat of runner stem. ISSR DNA fingerprint is simple, accurate and straightforward in certification of plant species. In term of extracts of the three species, TLC and HPLC methods can be employed although overlapping is still possible due to the similarity of the chemical profile. Therefore, further standardization of chemical methods to employ latest techniques such as LC-MS, is warranted to eliminate any potential adulteration.

Taken together, the combination of morphometric, genetic, and phytochemical methods with the aid of multivariate analysis, have consistently differentiated three *Centella* species in Australia, confirming their botanical classification and distribution in Australia. Since the grouping pattern based on ISSR molecular markers is consistent with their morphological characters, the DNA-based authentication technique provides an effective tool to identify the *Centella* species for quality control and research.

Although, *C. cordifolia* was genetically and chemically closer to *C. asiatica*, it had the highest triterpene glycosides, phenolics and antioxidant capacity, while *C. erecta* was distinctive from them. Furthermore, *C. cordifolia* is potentially a new source of *Centella* herb for medicinal purpose as it has a favorable high phenolic and saponin composition when compared to *C. asiatica*. This is the first comparative botanical study on *Centella* species, which provides a foundation for further systematic study on Centella.

## Author contributions

Conception, planning, and revision: GL; Experimental conducting, planning and writing: AA; Writing input and revision: J-LC, KW, KL and VR-N.

### Conflict of interest statement

The authors declare that the research was conducted in the absence of any commercial or financial relationships that could be construed as a potential conflict of interest.

## Supplementary material

Figure S2 Localities of the three *Centella* species, collected from different states in Australia. CA, *C. asiatica*; CC, *C. cordifolia*; and CE, *C. erecta*.
