# Supplementary figures and images for: Differentiation of Three Centella Species in Australia as Inferred from Morphological Characteristics, ISSR Molecular Fingerprinting and Phytochemical Composition
Source: Front Plant Sci. 2017 Nov 21;8:1980. doi: 10.3389/fpls.2017.01980 (PMC5702339; doi:10.3389/fpls.2017.01980)

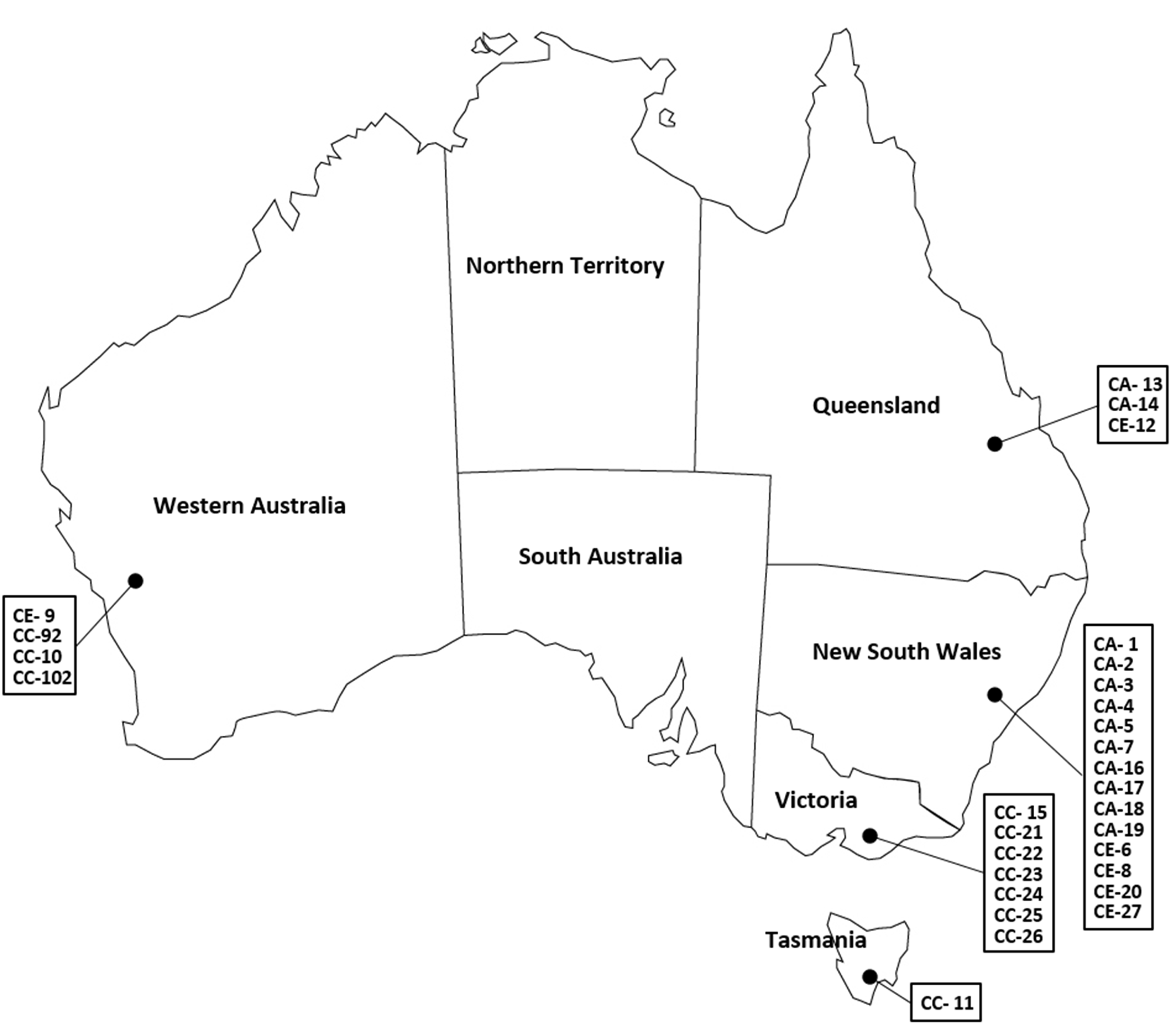

Supplement: Figure S2 — Localities of the three Centella species, collected from different states in Australia. CA, C. asiatica; CC, C. cordifolia; and CE, C. erecta. [file ImageS2.TIF]

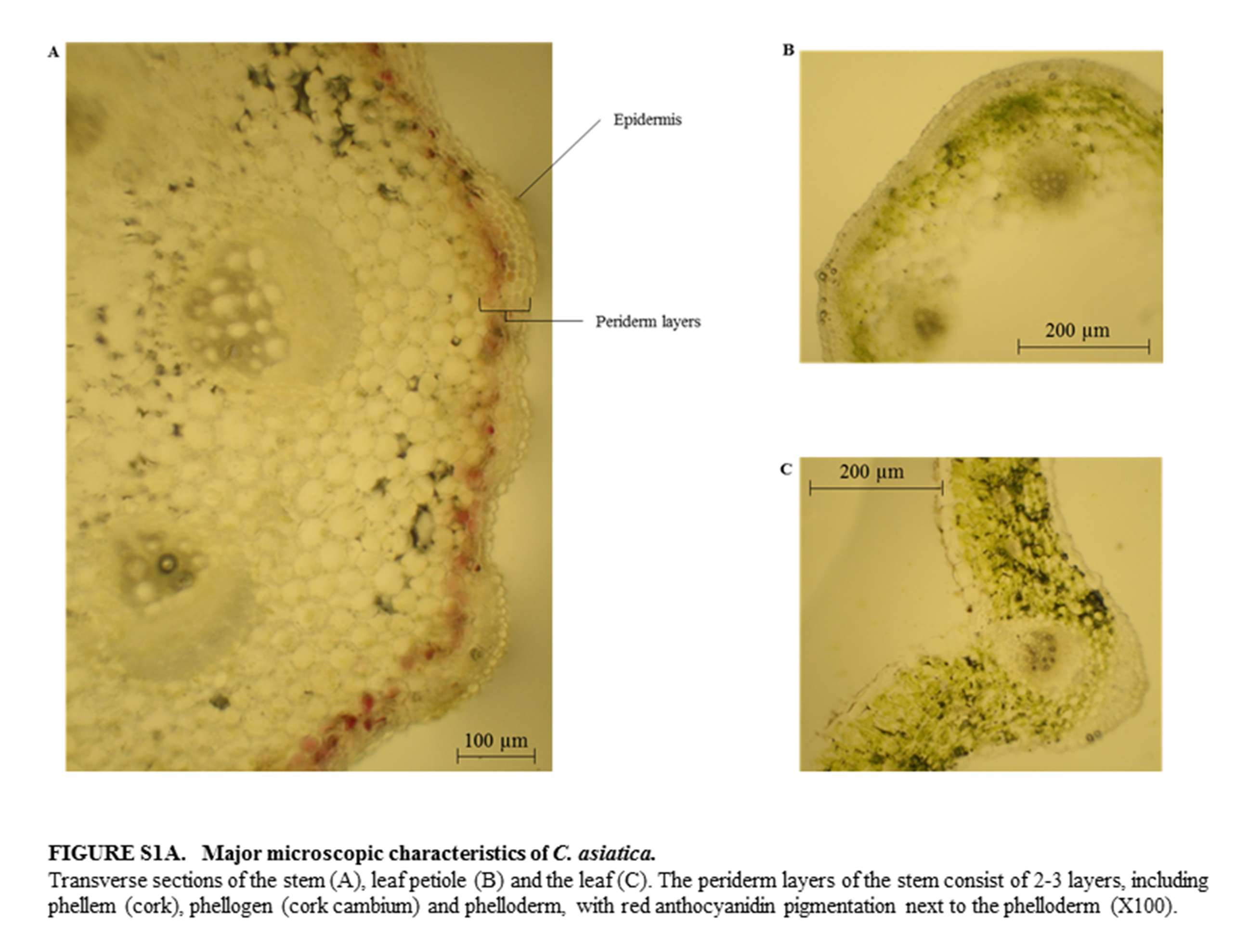

Supplement: Supplementary file 2 [file ImageS1a.TIF]

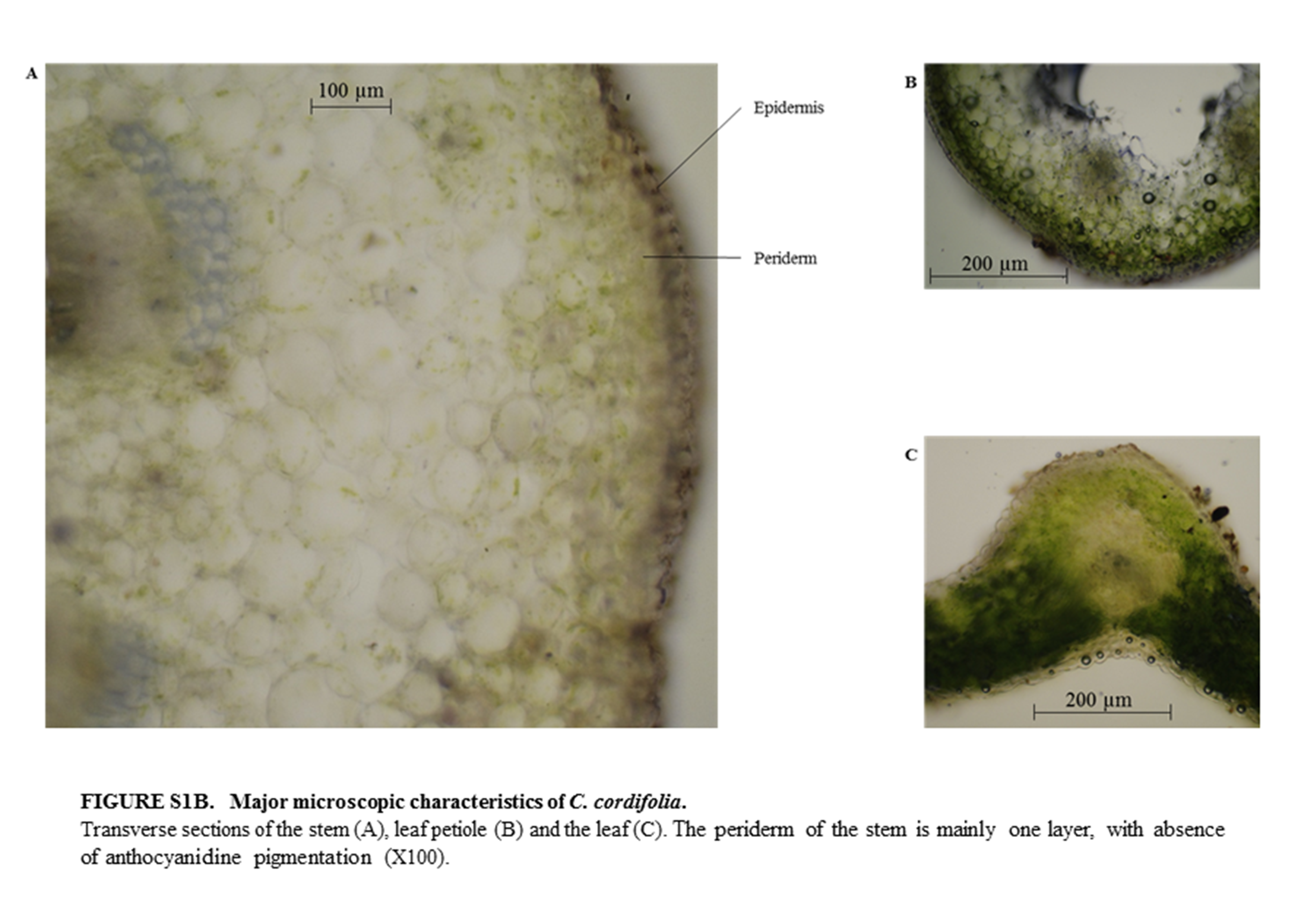

Supplement: Supplementary file 3 [file ImageS1b.TIF]

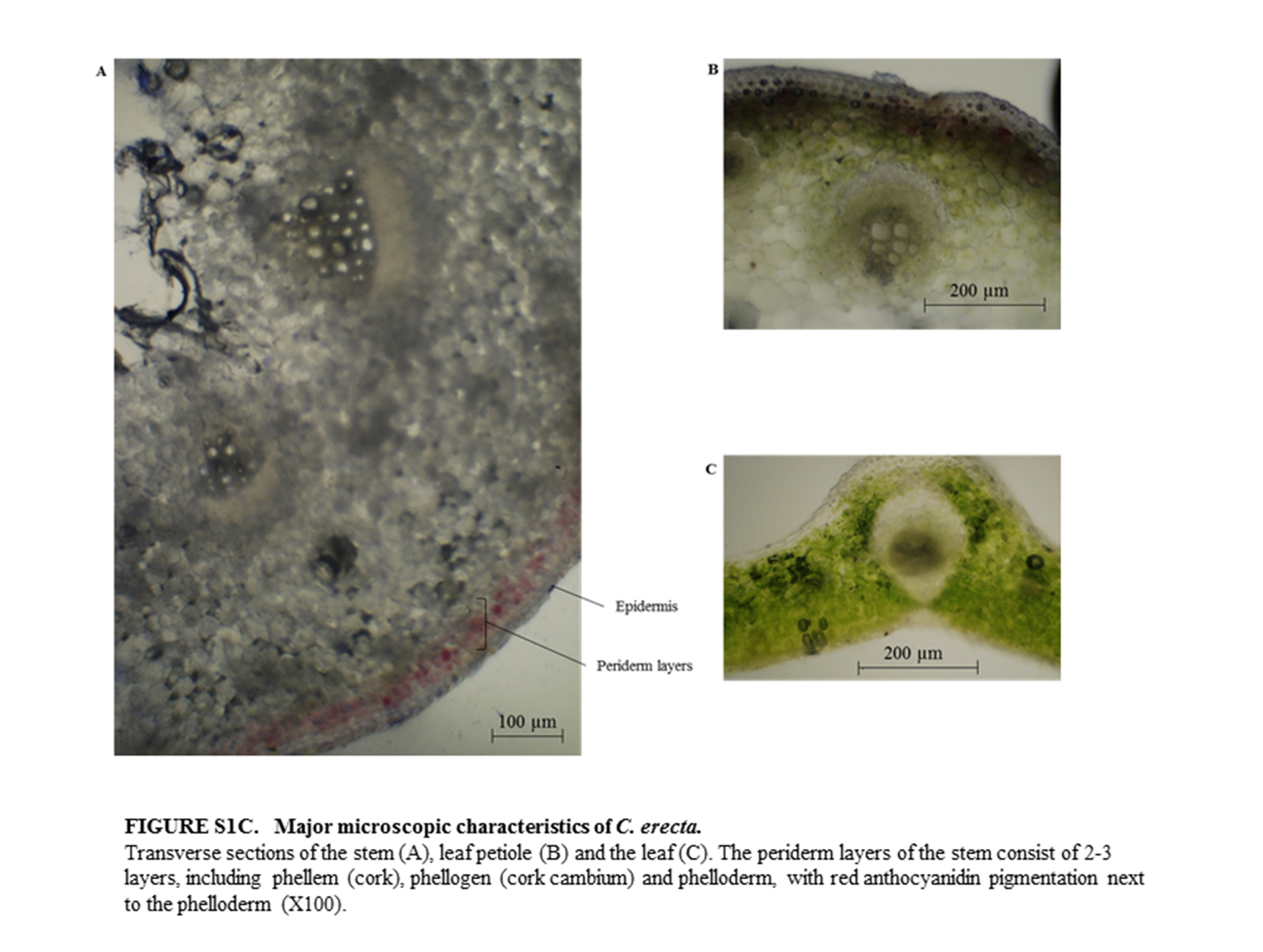

Supplement: Supplementary file 4 [file ImageS1c.TIF]
