# Supplementary material for: Differentiation of Three Centella Species in Australia as Inferred from Morphological Characteristics, ISSR Molecular Fingerprinting and Phytochemical Composition
Source: Front Plant Sci. 2017 Nov 21;8:1980. doi: 10.3389/fpls.2017.01980 (PMC5702339; doi:10.3389/fpls.2017.01980)
Supplement: Supplementary file 5 [file Table1.pdf]

TABLE S1. Correlation between the genetic diversity, morphological characters, TPC, TSC and antioxidant capacities based on Pearson’s coefficient.

	TPC	TSC	DPPH	ABTS	CUPRAC	Na	Ne	H	I	CL	TL	LSA	LBL	LBW	LBS	LMS	NDC	NPV	LP	CBP	AH	CF	SS	TS	CS
TPC	1																								
TSC	0.750(**)	1																							
DPPH	0.884(**)	.733(**)	1																						
ABTS	0.823(**)	.778(**)	.896(**)	1																					
CUPRAC	0.883(**)	.815(**)	.931(**)	.918(**)	1																				
Na	0.640(**)	.639(**)	.550(**)	.550(**)	.571(**)	1																			
Ne	.703(**)	.682(**)	.641(**)	.680(**)	.653(**)	.958(**)	1																		
H	.687(**)	.673(**)	.614(**)	.639(**)	.629(**)	.983(**)	.994(**)	1																	
I	.677(**)	.666(**)	.599(**)	.619(**)	.617(**)	.990(**)	.988(**)	.999(**)	1																
CL	.591(**)	.530(**)	.619(**)	.737(**)	.603(**)	.497(**)	.725(**)	.648(**)	.612(**)	1															
TL	.713(**)	.680(**)	.670(**)	.732(**)	.676(**)	.893(**)	.984(**)	.960(**)	.946(**)	.835(**)	1														
LSA	-.49	-.50	-0.3	-0.3	-.39	-.89	-.75	-.81	-.83	-0.1	-.63	1													

	9(**)	6(**)	37	25	9(*)	7(**)	5(**)	5(**)	8(**)	31	7(**)														
LBL	-0.337	-0.345	-0.169	-0.093	-0.232	-.760(**)	-.571(**)	-.646(**)	-.677(**)	0.098	-.431(*)	.929(**)	1												
LBW	-.550(**)	-.535(**)	-.379(*)	-.379(*)	-.432(*)	-.907(**)	-.783(**)	-.837(**)	-.857(**)	-0.192	-.675(**)	.985(**)	.905(**)	1											
LBS	-.433(*)	-0.333	-.443(*)	-.579(**)	-.419(*)	-0.217	-.472(**)	-.383(*)	-0.343	-.908(**)	-.610(**)	-0.107	-0.286	-0.038	1										
LMS	.404(*)	.488(**)	.448(*)	.414(*)	.465(*)	.677(**)	.631(**)	.654(**)	.662(**)	0.282	.576(**)	-.554(**)	-.425(*)	-.543(**)	0.145	1									
NDC	.524(**)	.580(**)	.531(**)	.510(**)	.543(**)	.753(**)	.750(**)	.758(**)	.760(**)	.461(*)	.717(**)	-.596(**)	-.441(*)	-.602(**)	-0.067	.937(**)	1								
NPV	-0.261	-0.308	-0.155	-0.176	-0.220	-0.355	-0.236	-0.282	-0.301	0.139	-0.153	.474(**)	.460(*)	.469(*)	-0.251	-0.245	-0.134	1							
LP	-.528(**)	-.479(**)	-0.309	-0.303	-.393(*)	-.695(**)	-.544(**)	-.604(**)	-.629(**)	0.024	-.428(*)	.861(**)	.832(**)	.874(**)	-0.194	-.387(*)	-.433(*)	.517(**)	1						
CBP	-0.268	-.439(*)	-0.298	-0.339	-.407(*)	-.516(**)	-.518(**)	-.522(**)	-.522(**)	-0.328	-.498(**)	.429(*)	0.284	.385(*)	0.061	-.638(**)	-.571(**)	0.247	0.131	1					
AH	-.726(**)	-.596(**)	-.633(**)	-.671(**)	-.633(**)	-.893(**)	-.953(**)	-.940(**)	-.932(**)	-.737(**)	-.950(**)	.677(**)	.503(**)	.721(**)	.505(**)	-.584(**)	-.702(**)	0.131	.537(**)	0.352	1				
CF	-.521(**)	-.450(*)	-.409(*)	-.440(*)	-.397(*)	-.721(**)	-.732(**)	-.735(**)	-.734(**)	-.482(**)	-.708(**)	.659(**)	.504(**)	.668(**)	0.325	-.396(*)	-.585(**)	-0.172	.485(**)	0.269	.736(**)	1			
SS	0.035	0.095	-0.083	-0.200	-0.044	.484(**)	0.213	0.315	0.359	-.519(**)	0.038	-.752(**)	-.848(**)	-.699(**)	.702(**)	.383(*)	0.277	-.490(**)	-.709(**)	-0.177	-0.137	-0.224	1		
TS	0.035	0.095	-0.083	-0.200	-0.044	.484(**)	0.213	0.315	0.359	-.519(**)	0.038	-.752(**)	-.848(**)	-.699(**)	.702(**)	.383(*)	0.277	-.490(**)	-.709(**)	-0.177	-0.137	-0.224	1.000(**)	1	

CS	0.223	-0.176	0.067	0.024	0.003	-0.016	0.064	0.035	0.022	0.231	0.110	0.099	0.106	0.047	-.392(*)	-0.354	-0.230	0.288	-0.092	.519(**)	-0.307	-0.193	-0.248	-0.248	1
